# Neglected Giant Scalp Tumors: A Report of Two Cases With an Analysis of Delay Factors

**DOI:** 10.7759/cureus.78456

**Published:** 2025-02-03

**Authors:** Kabita Kalita, Rishi K Gupta, Poresh Boruah, Jyotirmay Baishya, Nithin M

**Affiliations:** 1 Plastic and Reconstructive Surgery, Gauhati Medical College and Hospital, Guwahati, IND

**Keywords:** bcc, cm (cutaneous melanoma) bcc (basal cell carcinoma) scc (squamous cell carcinoma), fungating tumours, giant scalp tumours, healthcare seeking behaviour, psychosocial barriers, scalp tumours, scc, surgical reconstruction, tumor neglect syndrome

## Abstract

Tumor neglect is a concerning phenomenon in which patients delay seeking medical attention despite the presence of obvious malignant growths. This rare and largely unexamined issue involves patients disregarding tumors as a means of coping with the outward, visible signs of cancer. Here, we present two cases of extensively neglected scalp tumors: the first, a 40-year-old male from a socially isolated community with a 26 × 22 cm squamous cell carcinoma, neglected for 14 years due to his role as the sole breadwinner, geographical isolation in a hilly region, and deep-rooted cultural beliefs in traditional healing. The second case, a 42-year-old male, presented with a 22 × 20 cm undifferentiated pleomorphic sarcoma, with a four-year delay attributed to prior experience with a benign lesion, medical anxiety, and financial constraints. Both patients exhibited massive, fungating tumors requiring complex surgical management. This report analyzes the factors contributing to extreme tumor neglect and underscores the need to address psychosocial barriers to timely medical care.

## Introduction

Tumor neglect refers to a delay in seeking treatment despite visible or symptomatic growth, often driven by psychological, social, or systemic barriers. Studies indicate that nearly one-third of patients with evident tumors postpone medical consultation for more than three months [1]. This delay contributes to advanced disease progression, necessitating more complex surgical interventions and potentially leading to poorer outcomes [2].

Multiple factors influence tumor neglect, including psychosocial elements such as fear, denial, and cultural beliefs [3,4]. Socioeconomic constraints, including limited healthcare access, financial barriers, and inadequate health literacy, significantly affect patient decision-making [5]. Additionally, healthcare system-related challenges, such as complex referral pathways and limited availability of specialized care, further exacerbate delays [6].

Understanding the underlying causes of tumor neglect is essential for developing strategies that encourage early medical consultation and timely intervention. This case report presents two extensively neglected scalp tumors, illustrating the intricate interplay of these contributing factors. By examining these cases, we aim to enhance awareness of tumor neglect and emphasize the need to address barriers to prompt medical care.

## Case presentation

Case 1

**Figure 1:**
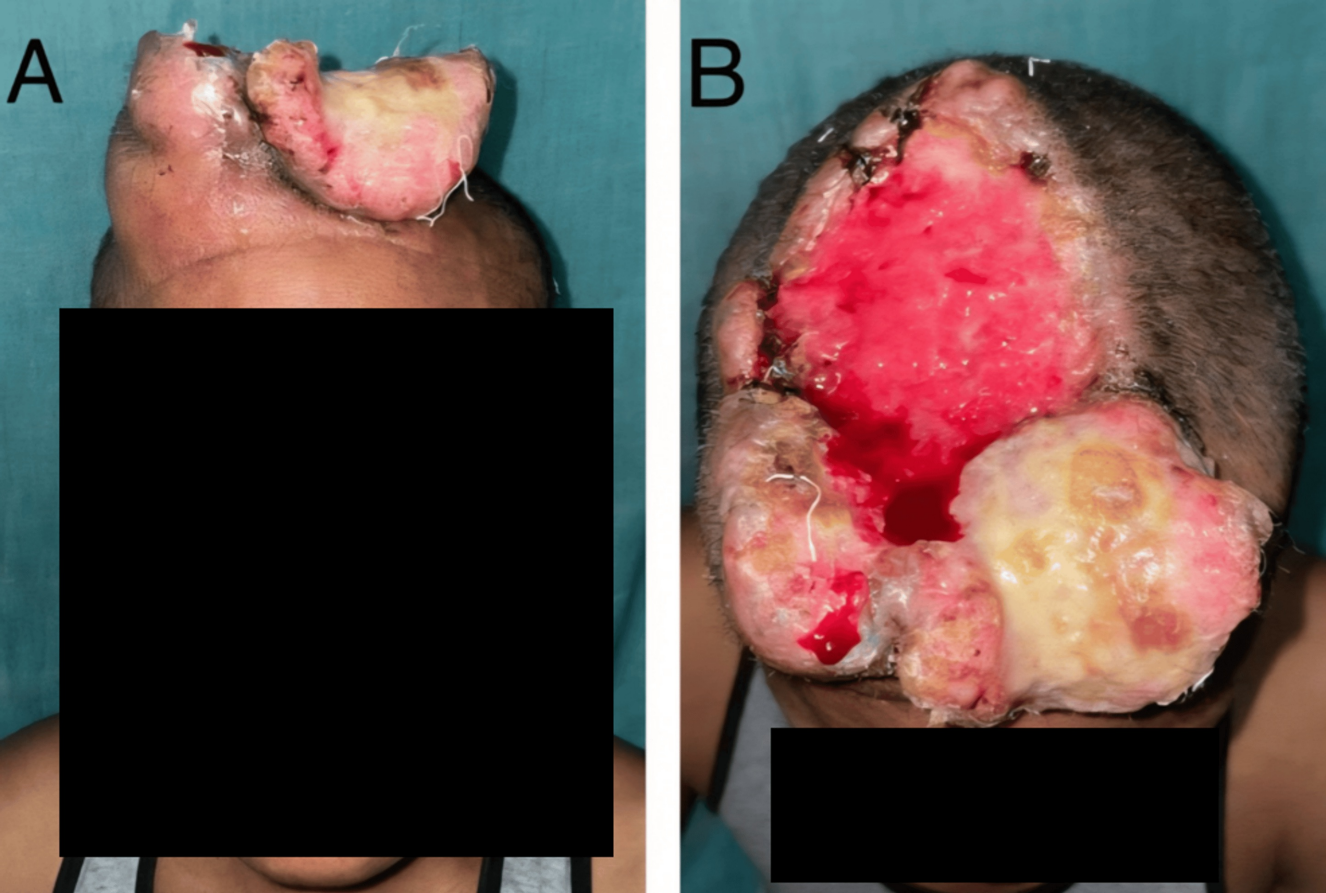
Case 1: (A) Anterior and (B) superior views of a fungating squamous cell carcinoma

A 40-year-old male coal mine worker from a very remote rural area presented with a 14-year history of a progressive scalp tumor. The patient belonged to a socially isolated community located in a hilly region. Physical examination revealed a foul-smelling and fungating mass measuring 26 × 22 cm, which covered most of the calvaria (Figure 1A). The tumor was irregular in texture, with both firm and friable areas, along with central necrosis. Vascularity was less prominent externally, with a few dilated veins over the forehead, although minor manipulation caused bleeding (Figure 1B). The patient reported mild intermittent dull aching pain, which had worsened over the last six months.

A biopsy was performed, confirming invasive squamous cell carcinoma. CT showed invasion into the outer table of the skull at the vertex, with cervical lymph node involvement; however, the node biopsy was negative for malignancy. A PET scan revealed no distant metastases.

**Figure 2:**
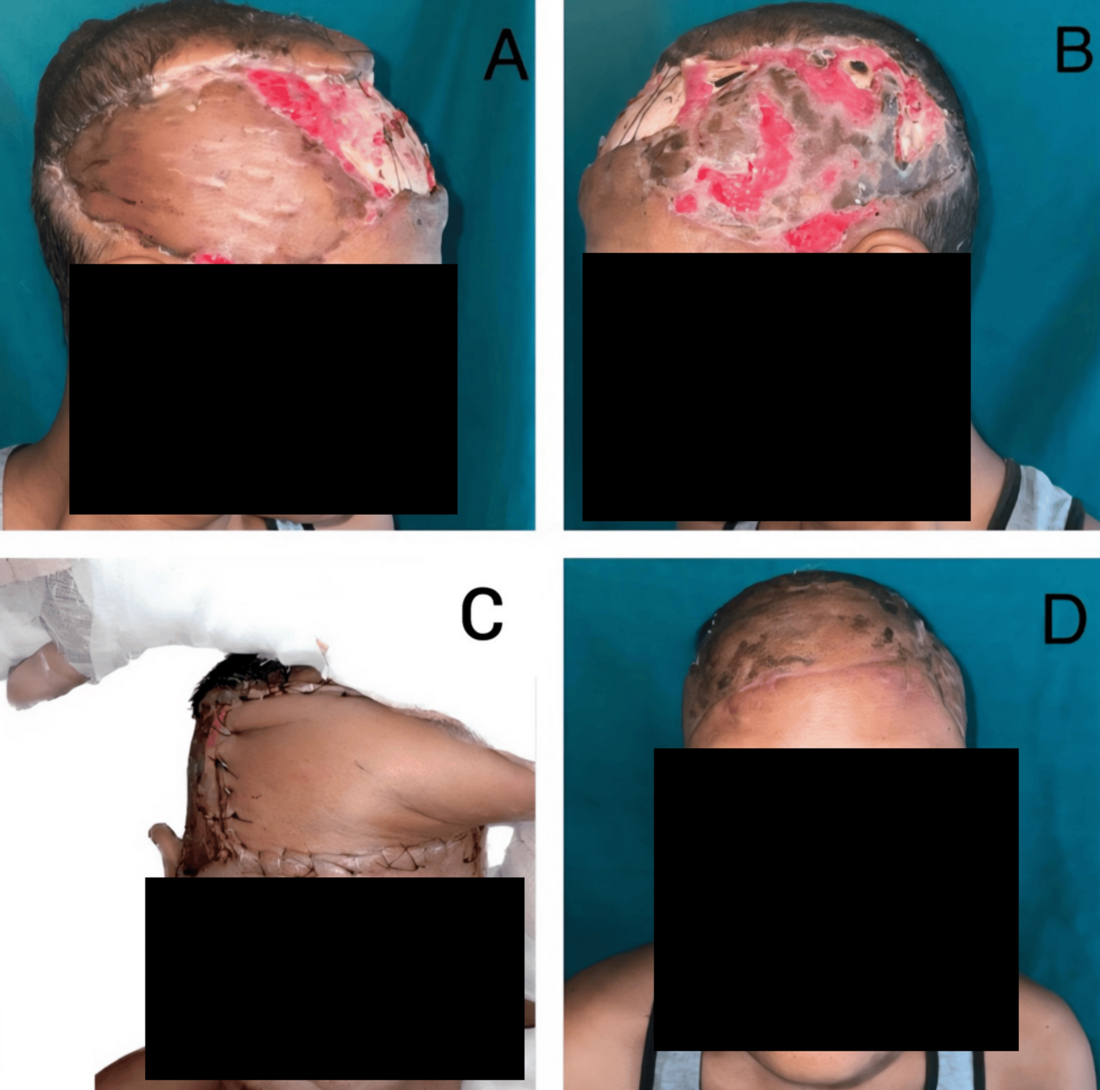
Case 1: (A, B) Post-first-stage surgery showing excision and local flap coverage. (C) Post-second-stage surgery with a pedicled radial forearm flap. (D) Final postoperative result

The patient underwent surgery in three stages. In the first stage, margin-free en bloc resection was performed, followed by reconstruction using local flaps from the surrounding scalp (Figure 2A, 2B). A two-stage radial forearm pedicled flap and split-thickness skin grafting were then performed (Figure 2C, Table 1). Histopathology confirmed moderately differentiated squamous cell carcinoma with clear margins, achieving a minimum peripheral clearance of 8 mm. The deep margin, at the level of the outer table of the skull, was also uninvolved.

**Table 1:** Clinical timeline for Case 1 and Case 2 SSG, split thickness skin grafting

Case	Event	Timeline	Details
Case 1	Onset of tumor	14 years ago	Progressive scalp tumors neglected due to geographic isolation and social factors
	Initial presentation	After 14 years	26 × 22 cm fungating mass diagnosed as invasive squamous cell carcinoma
	Diagnostic imaging	Upon presentation	CT showed skull invasion; PET scan showed no distant metastasis
	First surgery	Day 1	En bloc resection with local flap reconstruction and SSG over the scalp
	Second surgery - Stage 1	Day 14	First stage of radial forearm pedicled flap reconstruction
	Second surgery - Stage 2	Day 35	Detachment of radial forearm pedicled flap and SSG over the forearm donor site
	Postoperative radiotherapy	Three months post-op	Adjuvant therapy completed
	Follow-up	12 months post-op	Disease-free with no recurrence
Case 2	Onset of tumor	Four years ago	Recurrent lesion after prior benign tumor excision
	Initial presentation	After four years	22 × 20 cm fungating mass diagnosed as undifferentiated pleomorphic sarcoma
	Diagnostic imaging	Upon presentation	CT revealed skull and sagittal sinus invasion; no metastasis was detected
	Surgery	Day 1	Wide local resection with neurosurgical assistance; local flap and skin graft reconstruction
	Follow-up	Ongoing	Regular monitoring for recurrence

The patient experienced minor skin graft loss post-first surgery, which was subsequently regrafted during the second surgery. Adjuvant radiotherapy was administered, delivering a total dose of 60 Gy in 30 fractions over six weeks to the scalp and regional lymphatic drainage areas. This treatment aimed to reduce the risk of local recurrence due to the invasive nature of the squamous cell carcinoma. The patient experienced mild skin erythema and desquamation, which were managed with topical emollients. No significant late complications, such as radiation-induced necrosis, alopecia, or scarring, were observed during the 12-month follow-up. He remained disease-free at the 12-month follow-up (Figure 2D).

Case 2

**Figure 3:**
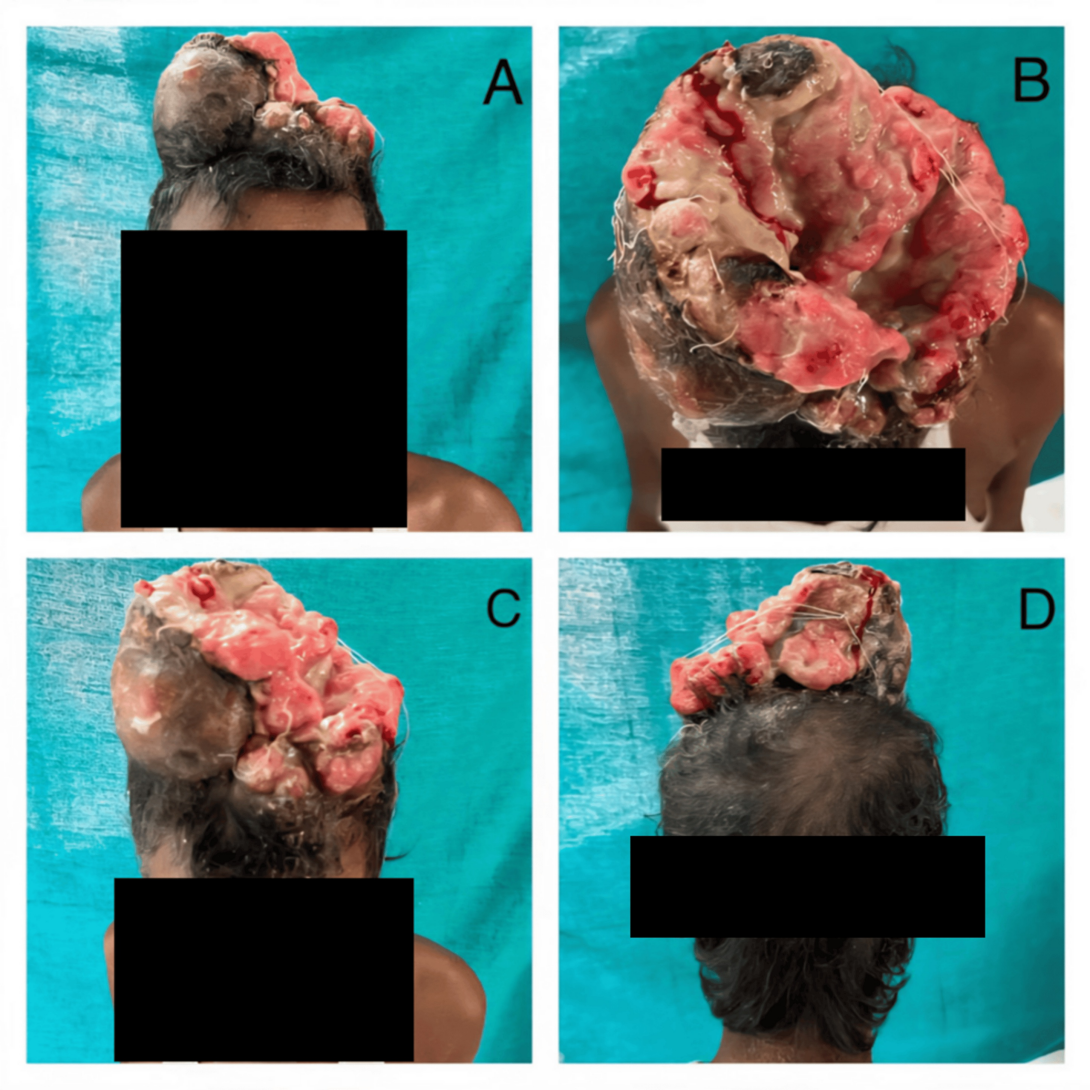
Case 2: (A) Anterior, (B) superior, (C) anterosuperior, and (D) posterior views of an undifferentiated pleomorphic sarcoma

A 42-year-old male presented with a four-year history of a progressive scalp tumor (Table 1). The patient had a history of surgical treatment for a smaller benign lesion in the same location five years earlier, with a one-year disease-free interval. However, a growth reappeared over the excised region, and the patient did not seek further treatment. Clinical examination revealed a foul-smelling, fungating mass measuring 22 × 20 cm (Figure 3A). The tumor exhibited heterogeneous consistency, with firm areas interspersed with softer regions. The surface was partially ulcerated, with pustular discharge and necrosis (Figure 3B, 3C, 3D). Vascularity was less prominent externally, but active bleeding suggested fragile internal vasculature. Initially, the patient experienced minimal pain, which progressed to deep, throbbing discomfort over the past year.

A biopsy was performed, suggesting a sarcomatoid lesion. CT revealed invasion of both the skull tables and involvement of the superior sagittal sinus at the vertex. No lymph node involvement or distant metastasis was detected on clinical or radiological examination.

**Figure 4:**
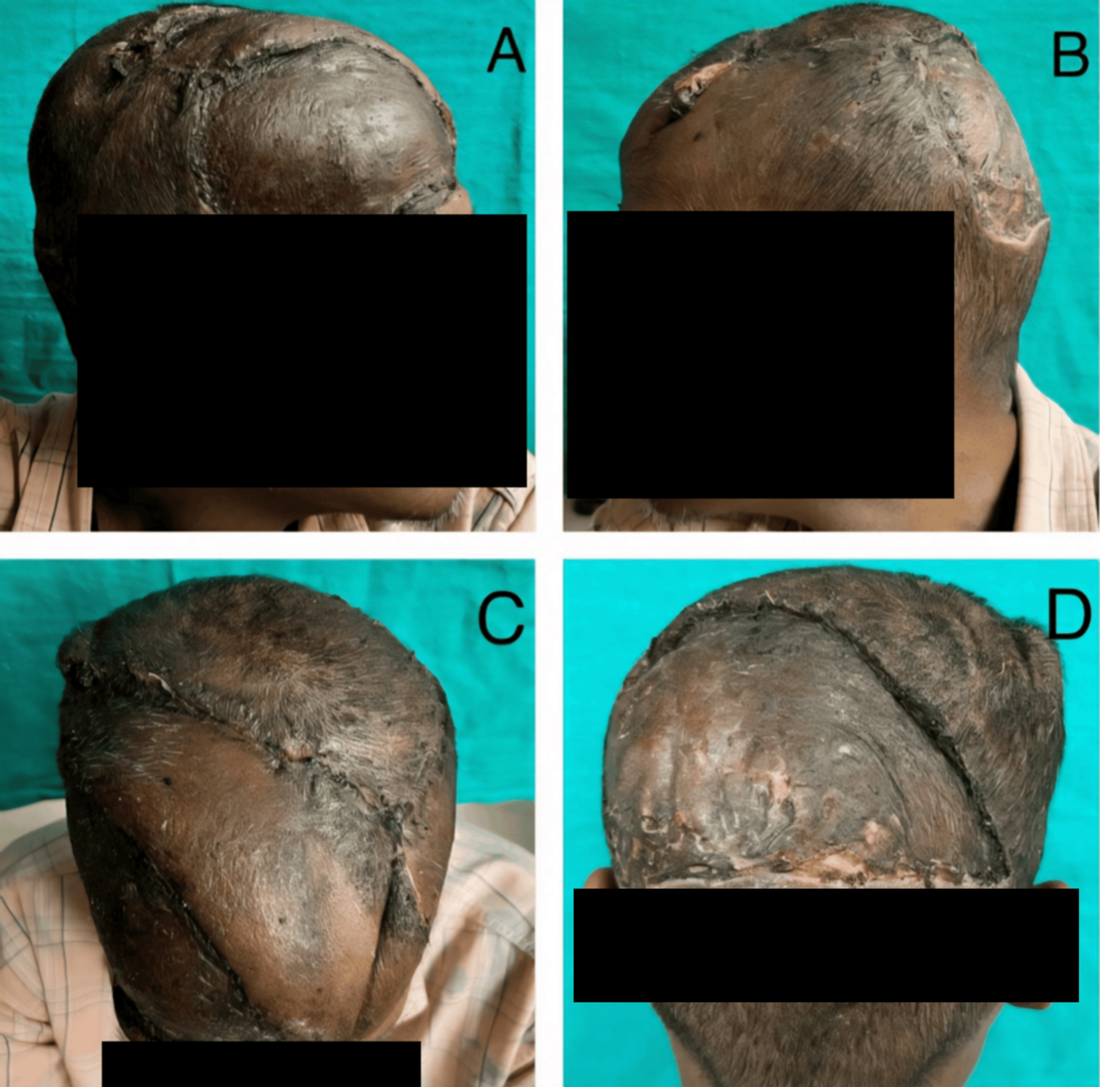
Case 2: (A, B) Postoperative lateral, (C) superior, and (D) posterior views of an undifferentiated pleomorphic sarcoma

The patient underwent multidisciplinary treatment, with surgery performed by the Department of Plastic Surgery in conjunction with the Department of Neurosurgery. He underwent wide local soft tissue resection, with careful dissection near the superior sagittal sinus, assisted by the neurosurgical team. Reconstruction was performed using a transposition flap from the parieto-occipital region of the scalp to cover the central defect, and split-thickness skin grafting was applied to the remaining areas (Figure 4A, 4B, 4C, 4D).

Histopathology revealed an undifferentiated pleomorphic sarcoma, with clear peripheral soft tissue margins of 5 mm and complete excision of the involved skull bone, confirming clear deep margins. The patient remains under regular follow-up for recurrent disease.

## Discussion

These cases exemplify the complex interplay of factors contributing to tumor neglect, a phenomenon that remains understudied despite its significant impact on patient outcomes. Our analysis reveals several key dimensions that warrant a detailed discussion.

In our first case, despite the obvious size and foul odor of the mass, the patient delayed seeking treatment primarily due to a combination of factors: his role as the sole breadwinner for his family, deeply rooted cultural beliefs in traditional healing, and a profound fear of job loss if he took time off for medical treatment. In the second case, the patient’s initial satisfactory experience with a benign lesion paradoxically led to a delayed presentation. He had developed a false sense of security about skin lesions, believing the new growth would also be benign and self-limiting. Additionally, medical anxiety stemming from his prior surgery, along with financial constraints, contributed to prolonged neglect. These psychological barriers, combined with a lack of awareness about malignant transformation, delayed medical attention.

The literature consistently reveals patterns in tumor neglect cases, with approximately one-third of patients with visible tumors delaying medical consultation for over three months [1]. This is particularly evident in malignancies of the skin and breast, where the visibility of the lesion paradoxically does not accelerate healthcare-seeking behavior [2]. Our cases, with delays of 14 years and four years, represent extreme examples of this phenomenon.

Common characteristics across reported cases include visible tumor locations, gradual growth, minimal initial symptoms, progressive local invasion, and relatively low metastatic rates despite extensive local disease [7,8].

Our cases share features with previously reported instances of extreme tumor neglect. Block et al. reported four cases of neglected tumors, including two scalp lesions with presentations similar to those in our patients [2]. Their series demonstrated that despite extensive local growth, these tumors often showed limited metastatic spread, as evidenced by biopsy and PET scans. This pattern suggests a possible immune-protective effect in long-standing tumor cases, although this hypothesis warrants further investigation [2,5].

Similar to cases reported by Varga et al., our patients exhibited distinct phases of neglect [8]. The first patient initially noticed a small scalp lesion (initial recognition phase) but attributed it to work-related scalp injuries, common among coal miners (minimization phase). He attempted treatment with local traditional medicines (self-management phase) until the tumor’s foul smell and bleeding began affecting his work and social life, ultimately prompting medical attention (complication-triggered presentation). The second patient followed a similar progression: he initially recognized the new growth at the previous surgery site (initial recognition) but dismissed it as another benign lesion, like his previous experience (minimization). He self-treated with over-the-counter medications and dressings (self-management) until the tumor’s size and skull involvement caused severe headaches and mobility issues, which finally led to medical presentation (complication-triggered presentation).

These progressions were influenced by multiple socioeconomic factors, including geographic isolation from healthcare facilities, limited access to healthcare resources, occupational constraints, transportation challenges, and healthcare expenses [9]. Healthcare system-related factors, such as the unavailability of specialized care, complexity of referral pathways, continuity of care issues, inadequate patient education systems, and communication barriers between providers and patients, further compounded these delays [3].

Psychosocial elements also play a critical role in tumor neglect. These include social isolation, limited support networks, cultural beliefs, reliance on traditional healing practices, and previous medical experiences (particularly relevant in our second case). Fear and denial mechanisms also significantly influence the delay in seeking care [10].

Recent research has identified specific psychological patterns observed in tumor neglect cases. Rabinowitz and Peirson described denial as a common initial coping mechanism, which can become maladaptive over time [4]. Denial, a typical human response to hardship, allows individuals to diminish the “threatening aspect of reality” to cope with difficult circumstances [11]. This pattern is evident in both of our cases, where patients were aware of their growing tumors but failed to seek timely medical care. This prolonged neglect significantly impacted the treatment approach, requiring complex multidisciplinary surgical planning and advanced reconstruction techniques. Despite no major complications in either case, neglected scalp tumors of this magnitude carry substantial risks, including intracranial extension leading to neurological deficits, massive hemorrhage, severe local infection with potential sepsis, and metastatic spread [12].

In Case 1, the squamous cell carcinoma’s proximity to major scalp vessels posed a risk of catastrophic bleeding, while Case 2’s tumor, involving the superior sagittal sinus, could have resulted in devastating venous complications or dural sinus thrombosis [13,14]. Other potential complications include chronic anemia due to tumor bleeding, severe local infection with osteomyelitis of the skull, and metastatic spread to regional lymph nodes or distant organs [15]. Early intervention would have reduced these life-threatening risks and simplified the surgical approach. These complexities align with the findings of Wax et al., who documented increased surgical challenges in neglected head and neck tumors [16].

The prevention strategies emerging from our cases and literature review include enhancing community health education, regular screening programs in isolated communities, and improved access to primary healthcare. Building trust between healthcare providers and isolated communities, along with the implementation of patient navigation programs, is also essential [6]. Healthcare professionals play a crucial role in the early identification of at-risk patients, recognition of psychosocial barriers to care, development of individualized intervention strategies, creation of supportive care environments, and facilitation of multidisciplinary care approaches [17].

In today’s healthcare landscape, the role of providers extends beyond clinical management. It encompasses addressing the underlying factors contributing to tumor neglect through a comprehensive approach that considers both the medical and psychosocial aspects of patient care. This approach is particularly relevant in communities with limited healthcare access.

## Conclusions

These cases highlight the complex interplay of factors contributing to delayed treatment-seeking behavior in tumor cases. In the first patient, socioeconomic constraints as the sole breadwinner, geographical isolation in a hilly region, and deeply ingrained cultural beliefs formed significant barriers to early medical intervention. In the second case, the patient’s previous experience with a benign lesion, combined with medical anxiety and financial constraints, led to prolonged neglect. Despite these delays, both cases were successfully managed through coordinated multidisciplinary surgical intervention, demonstrating that even extensively neglected tumors can be effectively treated with appropriate surgical approaches.

The successful surgical management of both cases underscores that even extensively neglected tumors can be treated effectively with the right multidisciplinary intervention. However, the complexity of the procedures, prolonged recovery periods, and increased risk of complications emphasize the importance of early detection and treatment. Future research should focus on understanding the psychological aspects of tumor neglect and developing targeted interventions to address healthcare-seeking delays. Healthcare providers should remain vigilant to potential barriers to care and create strategies to promote earlier patient presentation. This could include community outreach programs, improved patient education, and better access to healthcare services in remote areas.
